# Bcl-2 Immunoexpression in Feline Epitheliotropic Intestinal T-Cell Lymphomas

**DOI:** 10.3390/vetsci9040168

**Published:** 2022-03-31

**Authors:** Agustín Rebollada-Merino, Néstor Porras, Andrés Calvo-Ibbitson, Fernando Rodríguez-Franco, Antonio Rodríguez-Bertos

**Affiliations:** 1 VISAVET Health Surveillance Centre, Complutense University of Madrid, 28040 Madrid, Spain; agusrebo@ucm.es (A.R.-M.); nestorpo@ucm.es (N.P.); 2 Department of Internal Medicine and Animal Surgery, Faculty of Veterinary Medicine, Complutense University of Madrid, 28040 Madrid, Spain; ferdiges@vet.ucm.es; 3 VetPatólogos, Av. Isabel de Farnesio, 27, 28660 Boadilla del Monte, Spain; a.calvo@vetpatologos.com

**Keywords:** Bcl-2, epitheliotropic, feline lymphoma, oncoprotein, T-cell lymphoma

## Abstract

Lymphoma is the most common malignant hematopoietic neoplasm in domestic felines. Twenty-two cases of feline epitheliotropic duodenal T-cell lymphoma were characterized morphologically and immunohistochemically (CD3, *Pax5*, Ki-67), and Bcl-2 immunoexpression was established. Most cases were in domestic shorthair cats (88.2%), with a mean age of 11.2 years. All lymphomas were CD3+, with a low-to-moderate expression of Ki-67 (<30%). A correlation between the tumoral pattern of infiltration in the lamina propria and the intraepithelial distribution of the neoplastic lymphocytes was established (*p* = 0.0155). Intraepithelial nests of neoplastic lymphocytes were predominantly observed in lymphomas with a patchy distribution in the lamina propria, whereas intraepithelial plaques were seen in lymphomas with an obliteration pattern. Bcl-2 was expressed in neoplastic cells in all cases, and a higher expression was associated with increased villous stunting (*p* = 0.0221), and tended to be present in those cases with increased epithelial damage. The expression of Bcl-2 and the degree of epitheliotropism were correlated with neoplastic progression in epitheliotropic intestinal T-cell lymphomas; those displaying high Bcl-2 immunoexpression showed increased villous stunting and epithelial damage, suggesting that Bcl-2 is overexpressed in advanced tumor stages, and may be used as a predictor of tumoral behavior in feline epitheliotropic intestinal T-cell lymphomas. This entity showed many similarities with human MEITL, so the latter entity should be considered in further lymphoma classifications of domestic animals.

## 1. Introduction

Lymphoma is the most common malignant hematopoietic neoplasm in domestic felines [1,2,3,4]. Among the different forms, depending on the tumor location (multicentric, mediastinal, extranodal, or alimentary), alimentary lymphoma is considered the most prevalent [5,6,7,8]. Gastrointestinal lymphoma affects different sections of the gut—mainly the small intestine [8,9,10]—with a focal, multifocal, or diffuse distribution. Lymphoma can also involve the large intestine, local draining lymph nodes, liver, and spleen [3,7,8]. The course is often chronic and progressive, and clinical signs may or may not be present in the same way in different animals at the same disease stage. If present, these are often subtle, and include a progressive lack of appetite, lethargy, chronic diarrhea with or without melena, vomiting, and weight loss, leading to a poor general condition [8,9,11]. Sudden death may occur secondary to intestinal perforation and peritonitis [12].

Human intestinal T-cell lymphomas are subclassified into (1) enteropathy-associated T-cell lymphoma (EATL), (2) monomorphic epitheliotropic intestinal T-cell lymphoma (MEITL), (3) indolent T-cell lymphoproliferative disorder of the gastrointestinal tract (ITLPD), and (4) intestinal T-cell lymphoma, not otherwise specified (ITCL, NOS), according to the latest 2017 World Health Organization (WHO) classification [13]. The latest human WHO classification has not yet been validated for domestic animals. Human MEITL was formerly classified as type II EATL [3,14,15,16]. Feline type II EATL is believed to arise from the T lymphocytes homing in the intraepithelial compartment [2], and it is known to be the most frequent intestinal lymphoma subtype in cats [2,3,10,17].

Epitheliotropism, or the increased homing of neoplastic T-cells in the epithelium, is a dominant histopathological feature of human MEITL and, to a lesser extent, EATL [13,18]. However, in veterinary medicine, it remains unclear whether epitheliotropism is just a histological feature of type II EATL [3,7], epitheliotropic lymphomas are a subset of type II EATL [8], or epitheliotropic lymphomas should be classified as a separate entity [19]. Moreover, it should be considered that not all type II EATLs in cats display epitheliotropism [2,20,21]. Epitheliotropism is helpful to distinguish intestinal lymphomas from chronic lymphocytic inflammation [3,5,17]. However, in chronic inflammatory disorders there is also an increase in the number of intraepithelial lymphocytes. Therefore, epithelial tropism should not be employed alone to distinguish lymphomas from inflammatory enteropathies.

The sequential diagnostic approach for intestinal lymphoma diagnosis in domestic canines uses histology as the first step, followed by immunophenotyping, determination of the proliferation index by Ki-67 and, finally, polymerase chain reaction (PCR) for antigen receptor rearrangement (clonality test) as a confirmatory test in challenging cases [22]. The same approach has been proposed for feline intestinal lymphoma [17,23]. Ki-67 is used as a marker of cellular proliferation in canine and feline intestinal lymphomas, revealing significantly higher values compared with chronic inflammatory enteropathy [22,23], wherein it is considered a prognostic marker [22]. Despite this, Ki-67 should not be used alone to differentiate intestinal lymphoma from intestinal inflammatory disorders [22].

The B-cell lymphoma gene-2 (bcl-2) encodes the Bcl-2 protein—an anti-apoptotic factor that preserves the survival in non-proliferating cells, promoting DNA reparation [24,25,26,27]. The upregulation of this protein in some tumoral cells limits the activation of caspases, prevents cell death, and confers longevity to neoplastic cells [24,28]. This upregulation observed in some human lymphomas has paralleled recent research in veterinary medicine regarding Bcl-2 immunoexpression in feline lymphomas [24,25,26,28]. These studies showed that T-cell lymphomas were more likely to express this Bcl-2 oncoprotein compared with B-cell lymphomas [24,25,26,29,30]. Moreover, the expression of Bcl-2 transcripts in a feline lymphoma cell line (FT-1) was induced by some antineoplastic drugs, such as doxorubicin and prednisolone [28]. Bcl-2 expression is significantly higher in feline gastrointestinal lymphomas compared with cats with chronic inflammatory enteropathy [26]. Investigation of anti-apoptotic mechanisms in feline lymphomas may contribute to establishing effective therapeutic strategies for these tumors [28], and should be explored as a diagnostic marker to distinguish lymphoma from intestinal inflammatory disorders.

Here, feline epitheliotropic intestinal T-cell lymphomas were characterized histologically. The morphological parameters evaluated were correlated with the immunoexpression of Ki-67 and Bcl-2. Our results may contribute to understanding of the molecular mechanisms underlying the progression of feline epitheliotropic intestinal T-cell lymphomas.

## 2. Materials and Methods

Twenty-two feline intestinal T-cell lymphomas with epitheliotropism obtained from duodenal well-oriented endoscopic biopsies (*n* = 15), duodenal full-thickness biopsies (*n* = 2), and postmortem exams (*n* = 5) at the Veterinary Teaching Hospital of the Faculty of Veterinary Medicine (Complutense University of Madrid) and a private diagnostic laboratory (VetPatólogos) were included in this study. Complementary data—such as age, sex, breed, clinical history, and feline leukemia (FeLV) or immunodeficiency (FIV) virus status by ELISA test—were also recorded.

Samples were routinely processed for histology and stained with hematoxylin and eosin (HE). For each case, a histopathological study was evaluated following the World Small Animal Veterinary Association (WSAVA)’s International Gastrointestinal Standardization Group morphological criteria (villous stunting, epithelial injury, crypt distension, lacteal dilation, and mucosal fibrosis/desmoplasia), on a 0–3 scale [31].

The tumor infiltration pattern in the lamina propria (patchy, band, or obliteration) and the distribution of intraepithelial neoplastic lymphocytes (nests or plaques) were considered, as described in previous studies [2,22]. Regarding the tumor infiltration pattern, a patchy pattern was characterized as “discrete regions of increased lymphocyte density within the villous lamina propria”, a band pattern as “a band of increased lymphocyte density that spanned the crypt–villous junction”, and an obliteration pattern as a “complete lymphocytic infiltration of the villous and crypt lamina propria with the formation of a lymphocytic band beneath the crypt epithelium but above the muscularis mucosae” [2]. Regarding intraepithelial neoplastic lymphocyte distribution, nests were defined as “≥5 clustered intraepithelial lymphocytes”, and plaques were characterized by “≥5 adjacent epithelial cells overrun by lymphocytes” [22].

For immunohistochemistry, each formalin-fixed, paraffin-embedded sample of duodenum was cut into 4 µm serial sections, and the technique was performed as described elsewhere [32], and employing a commercial polymer-based detection system (Novolink Polymer Detection Systems, Leica, Germany) and diaminobenzidine as the chromogen. All antibodies used (Table 1) were pre-tested in human tissue, showing adequate immunoreaction.

Our rationale for antibody selection was as follows: CD3 (cluster of differentiation 3) is a protein complex and T cell co-receptor, *Pax5* (paired box 5) is the B-cell-lineage-specific activator protein (BSAP), and Ki-67 is a nuclear protein associated with cellular proliferation (expressed during G1, S, and G2, but absent in G0).

Lymph node tissue from a cat was used as a positive control for CD3 (paracortex), *Pax5* (germinal center), and Bcl-2 (mantle cell zone of follicles) [25,30], and intestinal tissue was used for Ki-67 (stem cells of the intestinal crypts). For negative controls, Tris-buffered saline solution was used instead of the antibody. The origin, dilution, and source of the antibodies employed are set out in Table 1.

Bcl-2 immunoreaction was evaluated as follows: Briefly, 100 lymphocytes were counted in 5 adjacent, non-overlapping fields under a high-power field (HPF) (400×) (500 cells in total), and immunostained cells were expressed as a percentage of the cell count. A score from 0 to 3 was assigned to each sample—grade 0 (absence): less than 10% immunopositive cells (Bcl-2 immunoreaction was considered positive if more than 10% positive cells, due to the low-level expression of this protein under physiological circumstances) [24,30]; grade 1 (mild): 10–30% immunopositive cells; grade 2 (moderate): 30–60% immunopositive cells; grade 3 (intense): more than 60% immunopositive cells. Ki-67 was evaluated as follows—grade 0 (absence): 0% immunopositive lymphocytes; grade 1 (mild): less than 15%; grade 2 (moderate): 15–30%; grade 3 (intense): more than 30%—based on previous studies in canine and feline intestinal lymphomas [22,23].

Statistical analysis was carried out using SAS statistics software 9.4. Fisher’s exact test was used to establish differences between a lamina propria neoplastic infiltration pattern and the intraepithelial distribution of neoplastic lymphocytes; the Kruskal–Wallis test was used to assess differences between a lamina propria neoplastic infiltration pattern and the rest of the parameters (i.e., Bcl-2 and Ki-67 expression, and morphological parameters); the Wilcoxon two-sample test was used to establish the differences between the intraepithelial distribution of neoplastic lymphocytes and the rest of the parameters, and Spearman’s correlation coefficients enabled statistical correlation of Bcl-2 expression, Ki-67 expression, and histopathological parameters. The statistical significance was set at *p* < 0.05.

## 3. Results

### 3.1. Clinical Findings

The age was known in 17 out of 22 animals, ranging from 3 to 16 years, with an average of 11.2 years. In those cases where the age was known, more than 88.2% were older than 10 years. There was the same sex distribution: 11 females and 11 males. Cats were domestic shorthairs (*n* = 21) and Siamese (*n* = 1). Clinical history reported included chronic vomiting (*n* = 6), weight loss (*n* = 5), chronic diarrhea (*n* = 4), anorexia (*n* = 3), intestinal obstruction (*n* = 2), bloody diarrhea (*n* = 1), acute pancreatitis (*n* = 1), and ascites (*n* = 1). Two out of 22 animals were positive for FeLV, and none was positive for FIV.

### 3.2. Histopathological Findings

The histopathological outcomes of the evaluation according to the parameters previously mentioned are shown in Table 2. Villous stunting was directly correlated with epithelial injury (*p* < 0.0001). Epithelial injury was more intense (grade 2–3) in those cases in which intraepithelial lymphocytes were organized in plaques; however, there was no statistical significance (*p* = 0.0657). Furthermore, mucosal (lamina propria) desmoplasia was directly correlated with villous stunting (*p* = 0.0046), epithelial injury (*p* = 0.0189), and crypt distension (*p* = 0.0331). Crypt distension and lacteal dilation were also correlated (*p* = 0.0045). The lamina propria infiltration pattern and intraepithelial distribution of neoplastic lymphocytes were statistically correlated (*p* = 0.0155); patchy distribution observed as multifocal aggregates of T-lymphocytes distributed in the luminal portion of the villi lamina propria was associated with the formation of intraepithelial nests, whereas almost all intraepithelial lymphocytes organized in plaques were observed in tumors with a lamina propria infiltration in the band and obliteration patterns.

### 3.3. Immunophenotyping

**Figure 1:**
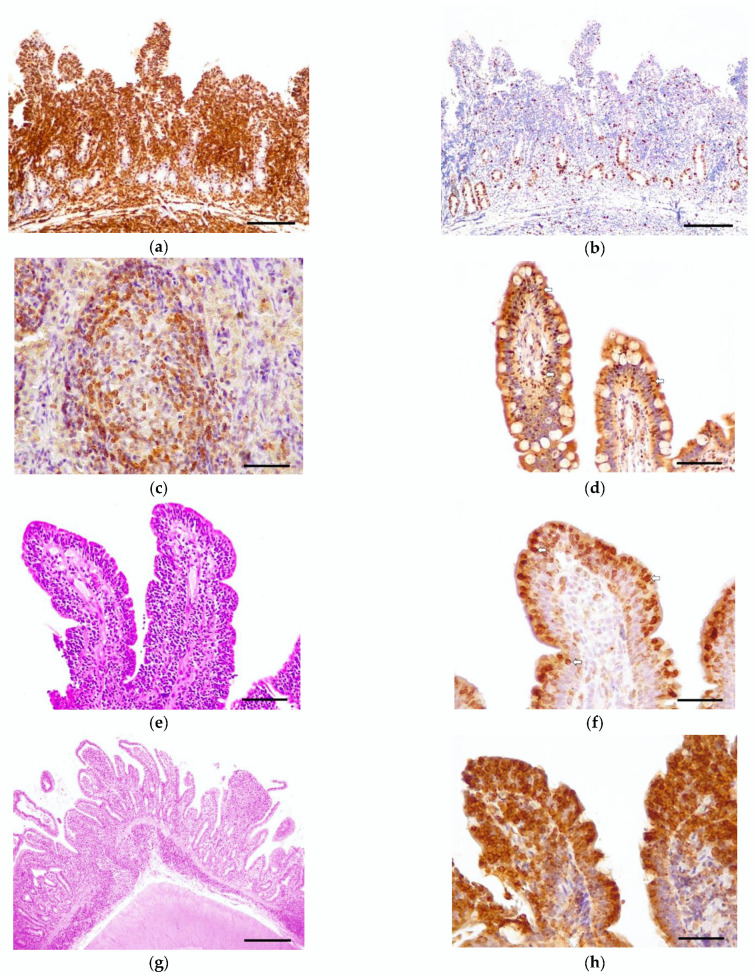
Feline epitheliotropic duodenal T-cell lymphoma, case 21, endoscopic biopsy: (**a**) The lamina propria is expanded by a monomorphic infiltration of T-lymphocytes. Exceptionally, the muscularis mucosae is shown, demonstrating transmural infiltration of neoplastic cells. Rabbit polyclonal anti-CD3 antibody. Scale bar: 1000 µm. (**b**) The neoplastic infiltration shows a moderate proliferation index. Proliferation is high in the stem cells of the crypts. Rabbit monoclonal anti-Ki-67. Scale bar: 1000 µm. (**c**) Bcl-2 positive control (feline mesenteric lymph node). Mantle cells of the lymphoid follicle immunoexpressed Bcl-2. Scale bar: 200 µm. (**d**) Case 2, endoscopic biopsy: Patchy pattern with formation of nests. Neoplastic lymphocytes, arranged in nests in the epithelium, showed a mild (grade 1) expression of Bcl-2 (arrows). Mouse monoclonal anti-Bcl-2 oncoprotein. Scale bar: 400 µm. (**e**) Case 20, endoscopic biopsy: Band pattern with formation of plaques. Intraepithelial lymphocytes were arranged in plaques and expanded into the lamina propria. Hematoxylin–eosin. Scale bar: 400 µm. (**f**) Case 20: Neoplastic T-cells display a moderate (grade 2) expression of Bcl-2—especially those located in the epithelium (arrows). Mouse monoclonal anti-Bcl-2 oncoprotein. Scale bar: 200 µm. (**g**) Case 14, necropsy sample: Obliteration pattern with formation of plaques. The neoplastic lymphocytes expanded and replaced normal tissue, causing severe villous stunting and crypt dilation. Hematoxylin–eosin. Scale bar: 1000 µm. (**h**) Case 14: Neoplastic cells organized in plaques and showing an intense (grade 3) Bcl-2 expression. Mouse monoclonal anti-Bcl-2 oncoprotein. Scale bar: 200 µm.

Positive control cells were positive for CD3 and *Pax5* in the cytoplasm, and for Ki-67 in the nucleus. The negative controls did not display an immunoreaction against any antibody. All tumors were diagnosed as T-cell lymphomas (22/22), since they expressed the CD3 immunophenotype, and according to the neoplastic lymphocytes’ distribution (Figure 1a). *Pax5* was expressed in scattered cells in the lamina propria, especially in the tumor periphery. Ki-67 immunoexpression in lymphocytes, diffusely observed in the crypts of the epithelium, was low (grade 1) in 17/22 cases, and moderate (grade 2) in 5/22 (Figure 1b).

### 3.4. Bcl-2 Immunoexpression

**Figure 2:**
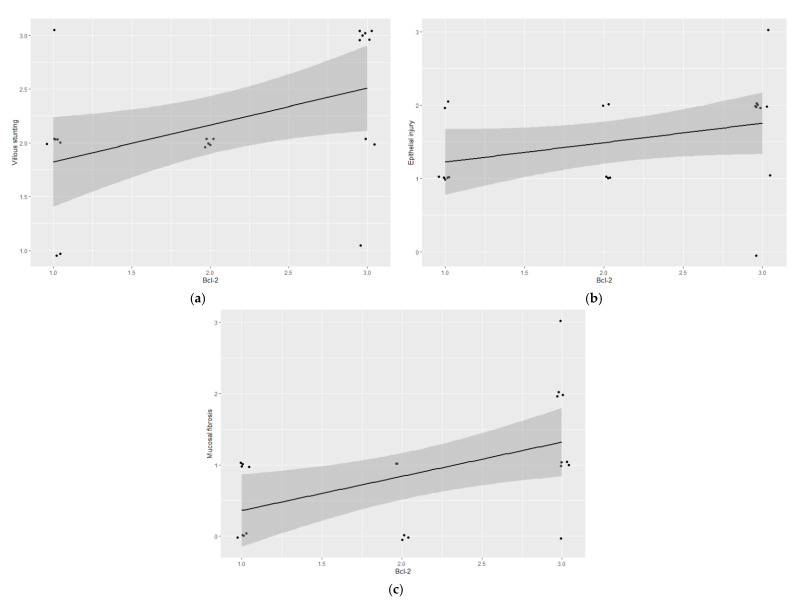
Feline epitheliotropic duodenal T-cell lymphoma: Plots of Spearman’s correlation coefficients between Bcl-2 and (**a**) villous stunting, (**b**) epithelial injury, and (**c**) mucosal desmoplasia.

Feline lymph node controls showed a cytoplasmic and membranous expression of Bcl-2 in the mantle cells (Figure 1c). All of the epitheliotropic intestinal T-cell lymphomas in this study showed Bcl-2 immunoreaction (22/22). Eight cases were included in low expression (grade 1) (Figure 1d), five in moderate (grade 2) (Figure 1e,f), and nine in intense (grade 3) (Figure 1g,h). Bcl-2 immunoexpression was statistically associated with villous stunting (*p* = 0.0221) (Figure 2a)—the higher the expression of Bcl-2, the more intense the villous stunting. Similarly, the cases with increased expression of Bcl-2 tended to display higher epithelial injury despite no significant statistical correlation (*p* = 0.0591) (Figure 2b). Mucosal desmoplasia was associated with Bcl-2 expression (*p* = 0.0159) (Figure 2c): low-to-moderate expression was correlated with the absence of (or mild) desmoplasia, whereas a higher expression was associated with mild-to-moderate desmoplasia. The correlation between Bcl-2 and Ki-67 showed no significance (*p* > 0.05). Despite this, in tumors with a moderate proliferation index (15–30% Ki-67 immunoexpression), the Bcl-2 expression was intense in 4/5 cases.

## 4. Discussion

The duodenum is the third most commonly affected region in feline intestinal lymphoma [3]. Duodenal effects can also occur in a primary form, or as a part of the disease’s progression toward the intestinal tract. In this study, only duodenal endoscopic biopsies were assessed, because of their higher accessibility with upper intestinal endoscopy [26], and because they have demonstrated diagnostic potential in previous cases [8,10,11]. Despite the fact that we employed a limited sample size due to the strict inclusion criteria, our study included more endoscopic biopsies than previous reports [26], which may make it more applicable from a routine diagnostic point of view.

Feline epitheliotropic intestinal lymphomas of T-cell origin have, to date, been included in type II EATL according to the latest WHO Classification of Hematopoietic Neoplasms in Domestic Animals [2,3,10]. Interestingly, not all feline type II EATLs have been reported to display epitheliotropism [2]. As epitheliotropism of neoplastic lymphocytes is one of the main inclusion criteria for MEITL (formerly type II EATL) in human beings, this leads us to believe that there are feline intestinal T-cell lymphomas being incorrectly diagnosed as feline type II EATL. In fact, some authors have suggested that most feline intestinal T-cell lymphomas today diagnosed as type II EATL would better fit into the human MEITL or ITLPD categories due to their morphological and prognostic similarities [21,33]. A novel adaptation of the WHO’s feline lymphoma classification could lead to the definition of appropriate morphological criteria for accurate diagnosis. Proper classification of feline intestinal lymphomas would permit the determination of prognostic and survival differences between subtypes. Therefore, different anti-neoplastic therapies could be targeted against specific subtypes, thus significantly improving prognosis and disease management in domestic cats.

Epitheliotropic intestinal T-cell lymphomas in human beings are derived from the intraepithelial lymphocytes, and are classified as MEITLs [14,16]. They are composed of a small-to-medium monomorphic population of T-cell lymphocytes, as described in the present study, and previously reported in feline epitheliotropic intestinal lymphomas [19]. Epitheliotropism is always evident, and the neoplasia progresses aggressively, causing crypt destruction and villous broadening and stunting [16]. Although villous blunting and fusion have been previously reported in human and feline epitheliotropic intestinal lymphomas [2,16,19,33], in this survey we correlated villous stunting with epithelial injury in feline intestinal lymphomas for the first time. Herein, we observed that the epithelial damage was greater when plaques were observed in the epithelium, indicating a higher tissue disruption as epitheliotropic lymphoma progresses. To the authors’ knowledge, this is the first report that establishes a correlation between the pattern of tumoral infiltration in the lamina propria and the intraepithelial distribution of neoplastic lymphocytes in epitheliotropic lymphomas of the intestine. Intraepithelial nest formation was predominantly observed in those lymphomas with patchy (multifocal) distribution in the lamina propria of the villi, whereas intraepithelial plaques were seen in those cases with a higher degree of effect on the mucosa—mainly in an obliteration (diffuse) pattern. This suggests that the intense clustering of lymphocytes is related to tumor progression, as higher aggregates of intraepithelial neoplastic lymphocytes lead to expansion of the tumor.

The mitotic count in epitheliotropic intestinal lymphomas of humans is higher compared with other subtypes of intestinal T-cell lymphoma [14], which, together with tissue disruption and transmural invasion, justifies their poor prognosis in human beings [14,16]. Interestingly, we have observed that feline epitheliotropic intestinal T-cell lymphoma cases with a proliferation index higher than 15% display an intense Bcl-2 immunoreaction in most cases, although the limited sample size precludes a definitive conclusion. This suggests similarities with human MEITL, as described by Wolfesberger *et al*. [21]. Furthermore, our results contrast with the low mitotic rate reported in feline type II EATL, which is known to be a slow-progressing and indolent tumor [21]. Some feline epitheliotropic lymphomas have been reported to display a reduced mitotic count with hematoxylin–eosin [21]. However, many studies did not assess the proliferation index employing Ki-67, and may thus have misidentified the proliferation index in the tumor. This suggests the importance of making use of this non-histone protein together with immunophenotyping in an early approach to distinguish epitheliotropic intestinal T-cell lymphomas from other intestinal lymphomas and chronic enteropathies in cats [23], as has been proposed in dogs [22].

The molecular mechanism beyond the proliferation of neoplastic lymphocytes in the villous epithelium that leads to the establishment of intestinal lymphoma remains to be completely understood. The overexpression of Bcl-2 protein in some human and feline lymphomas suggests that the dysregulation of apoptosis could be one of the main molecular mechanisms underlying neoplastic cell transformation and progression, thus being correlated with a poor prognosis [27,30,34]. In fact, Bcl-2 was reported to be overexpressed in intestinal adenomas, precluding carcinogenesis and their transformation into carcinomas in mice and human beings [35]. Feline lymphomas—particularly those arising from T lymphocytes—were more likely to show high Bcl-2 expression via immunohistochemistry in several studies [24,25,26,30]. Additionally, the expression of Bcl-2 in feline lymphoma T-cell lines has also been reported [28,29]. The quantification of neoplastic lymphocytes expressing Bcl-2 has allowed us to categorize cases and correlate them with the morphological features. High expression of Bcl-2 in neoplastic lymphocytes was statistically correlated with increased villous stunting, and tended to be presented in those cases with increased epithelial damage, suggesting that there is a loss of inhibition of the Bcl-2 oncoprotein in advanced tumor stages associated with epithelial disruption, which may worsen prognosis. Human T-lymphoblastic lymphoma/leukemia cells have shown to overexpress Bcl-2, accelerating malignant transformation by suppressing Myc-induced apoptosis [36].

Some authors have reported increased desmoplasia in almost half of cases of feline low-grade intestinal T-cell lymphoma [33]. Here, we found a direct statistical correlation between Bcl-2 expression and mucosal desmoplasia, which may be associated with the activation and differentiation of fibroblasts by the Bcl-2 protein [37]. Desmoplasia should be considered a negative factor during the histological assessment of feline epitheliotropic intestinal T-cell lymphomas. In fact, desmoplasia in the gastrointestinal tract alters digestion and absorption functions and has been reported to be a cause of mortality in human inflammatory bowel disease [37]. Interestingly, it has recently been reported that a Bcl-2 antagonist prevents fibrosis in a murine model of chronic colitis [37]. Future therapeutic approaches targeting Bcl-2 could reduce desmoplasia in feline epitheliotropic intestinal T-cell lymphomas and, therefore, may contribute to improve prognosis and survival in cats.

Progression from inflammatory bowel disease to lymphoma in cats has been widely suggested. In fact, the early expression of Bcl-2 in feline inflammatory enteropathy suggests a transition between inflammatory and neoplastic disorders [24], but this has not been proven [26]. In the authors’ opinion, prospective studies are needed to establish a solid correlation in this transition from inflammatory enteropathies to some subtypes of intestinal lymphoma in cats, which is highlighted by the fact that tumor behavior may vary in different animals due to environmental or genetic factors. Oncoprotein expression could be promising in the further assessment of malignant progression from feline inflammatory enteropathies to intestinal lymphoma.

## 5. Conclusions

The findings presented herein are intended to be valuable in the classification of feline lymphoma subtypes, and to provide molecular insights into the development and progression of epitheliotropic intestinal T-cell lymphoma in cats. Herein, feline epitheliotropic duodenal T-cell lymphomas showed many similarities with human MEITL, so the latter entity should be considered in further lymphoma classifications of domestic animals. Further prospective studies assessing clinical behavior and survival differences between feline epitheliotropic intestinal T-cell lymphomas and other T-cell intestinal lymphoma subtypes are needed to establish prognostic differences of this entity in cats, like those that currently exist for human beings.

## Figures and Tables

**Table 1:** Antibodies employed, dilution, and source.

Antibody	Dilution	Source
Mouse monoclonal anti-Bcl-2	1:100	Dako-Agilent
Rabbit polyclonal anti-CD3	1:100	Dako-Agilent
Mouse monoclonal anti-*Pax5*	1:25	Dako-Agilent
Rabbit monoclonal anti-Ki-67	1:300	Novus Biologicals

**Table 2:** Feline epitheliotropic duodenal T-cell lymphomas: Results of the histopathological scoring according to WSAVA-proposed parameters and Ki-67 and Bcl-2 immunoexpression.

Case	Lamina Propria Pattern	Intraepithelial Distribution	Villous Stunting	Epithelial Injury	Crypt Distension	Lacteal Dilation	Mucosal Desmoplasia	Bcl-2	Ki-67
1	Patches	Plaques	2	1	2	2	1	1	1
2	Patches	Nests	1	1	3	1	0	1	1
3	Patches	Nests	2	1	2	3	1	2	1
4	Band	Nests	2	1	1	0	0	3	1
5	Band	Plaques	3	3	2	2	3	3	2
6	Patches	Nests	2	1	2	2	0	1	2
7	Band	Plaques	3	2	2	3	2	3	1
8	Obliteration	Plaques	1	0	1	1	1	3	2
9	Patches	Plaques	2	1	0	1	0	2	1
10	Obliteration	Plaques	2	1	2	2	1	1	1
11	Obliteration	Plaques	2	2	2	1	2	3	1
12	Obliteration	Plaques	3	2	2	3	2	3	1
13	Obliteration	Plaques	2	2	1	1	1	2	1
14	Obliteration	Plaques	3	2	2	2	1	3	2
15	Obliteration	Plaques	3	2	1	0	1	3	2
16	Band	Plaques	2	2	0	0	0	1	1
17	Band	Plaques	1	1	1	1	0	1	1
18	Obliteration	Plaques	2	1	1	1	1	1	1
19	Obliteration	Plaques	3	2	2	2	1	1	1
20	Band	Plaques	2	1	1	1	0	2	1
21	Obliteration	Plaques	3	2	3	2	1	3	1
22	Obliteration	Plaques	2	2	1	3	0	2	1

## Data Availability

Data are contained within the article or Supplementary Materials.
